# Unfolded RPCA Network for Mitigating Inter-Transmitter Code Interference in MIMO PMCW Systems

**DOI:** 10.3390/s26113316

**Published:** 2026-05-23

**Authors:** Yonghee Lee, Jong-Ho Lee, Seongwook Lee

**Affiliations:** 1 Department of Electrical and Electronics Engineering, College of ICT Engineering, Chung-Ang University, Dongjak-gu, Seoul 06974, Republic of Korea; yhl0114@cau.ac.kr; 2 School of Electronic Engineering, Soongsil University, Seoul 06978, Republic of Korea

**Keywords:** inter-transmitter code interference, multiple-input and multiple-output (MIMO), phase-modulated continuous-wave (PMCW) systems, robust principal component analysis (RPCA)

## Abstract

Phase-modulated continuous wave (PMCW) has emerged as a promising waveform candidate for next-generation integrated sensing and communication systems due to its favorable sensing performance and multiplexing capability. In multiple-input and multiple-output (MIMO) PMCW systems, fast-time code-division multiplexing enables simultaneous transmission from multiple transmitters but causes inter-transmitter code interference due to non-ideal cross-correlation properties. The interference is observed to manifest as a low-rank component in the range–Doppler domain while target echoes appear as sparse components. This structural distinction motivates the use of robust principal component analysis (RPCA) for interference mitigation. In practice, conventional RPCA incurs high computational complexity due to the singular value decomposition (SVD) required at every iteration. To address this limitation, we propose an unfolded RPCA network in which each iterative step is mapped to a network stage and SVD is replaced by a factorized low-rank approximation. The proposed network also incorporates stage-wise learnable parameters for adaptive interference mitigation in MIMO PMCW systems. Simulation results demonstrate that the proposed method achieves interference mitigation performance comparable to conventional RPCA with 21.2 times lower inference latency. These results confirm the effectiveness and computational efficiency of the proposed method for real-time mitigation of inter-transmitter code interference in MIMO PMCW systems.

## 1. Introduction

Radar systems provide robust detection performance under varying illumination and adverse weather conditions and have become a key sensing modality across a wide range of applications, including advanced driver assistance systems, autonomous driving, and synthetic aperture radar (SAR)-based remote sensing [1,2,3]. Recently, integrated sensing and communication (ISAC), which enables sensing and communication functions within a single system, has emerged as a core technology for next-generation wireless systems [4]. In particular, the importance of multiple-input and multiple-output (MIMO) technology has attracted growing attention because it can achieve high sensing performance and spectral efficiency with limited hardware resources [5]. To satisfy these requirements, various radar waveforms have been investigated for MIMO ISAC systems [6].

Among the candidate waveforms, frequency-modulated continuous wave (FMCW) has been widely adopted in MIMO radar systems due to its relatively simple hardware implementation and mature signal-processing framework [7]. However, FMCW was originally designed as a radar-centric waveform and offers limited flexibility for joint waveform design and integration with communication systems [8]. To overcome this limitation, phase-modulated continuous-wave (PMCW) waveforms based on sequence spreading and multicarrier waveforms such as orthogonal frequency-division multiplexing (OFDM) have attracted considerable attention as promising candidates for ISAC because of their compatibility with communication signal structures [9]. Specifically, OFDM can leverage its multicarrier structure to simultaneously carry communication data and perform sensing [10]. Nevertheless, OFDM suffers from several drawbacks, including high peak-to-average power ratio (PAPR), amplifier nonlinearity, and sensitivity to intercarrier interference caused by Doppler spread [11]. By contrast, PMCW is well matched to the code-sequence structure commonly used in digital communications [12]. It also offers several advantages for radar applications, including relatively low PAPR, robustness against interference due to code spreading gain, and high range resolution [13,14]. In particular, a fast-time code-division multiplexing (CDM)-based MIMO PMCW system assigns different codes to different transmitters and enables simultaneous transmission over the same time–frequency resources [15]. This property allows separation of signals from multiple transmitters and virtual array formation simultaneously, which leads to improved sensing performance.

Despite these advantages, interference mitigation remains a major challenge in radar signal processing, and various signal processing-based and deep learning-based approaches have been investigated to suppress interference and improve detection performance [16,17,18,19,20]. In fast-time CDM-based MIMO PMCW systems, inter-transmitter code interference is a key challenge because multiple transmitters share the same time–frequency resources and operate simultaneously [21]. The non-ideal cross-correlation properties of the assigned codes generate residual interference components, which raise the interference floor and degrade target detection performance. To mitigate this problem, a compressive sensing-based greedy algorithm was proposed in [21] for suppressing inter-transmitter interference in MIMO PMCW systems.

Meanwhile, inter-transmitter code interference in MIMO PMCW systems appears as a structured component distributed over a wide region in the range–Doppler (RD) domain, whereas target echoes exhibit sparse characteristics and remain localized within a limited region. In other words, the interference component can be viewed as a spatially correlated background component, while the target echoes can be viewed as locally supported sparse components. This structural distinction naturally motivates the use of robust principal component analysis (RPCA), which decomposes an observation into a low-rank component and a sparse component [22]. RPCA has been widely used in computer vision for separating low-rank background and sparse foreground components [23,24,25]. Its applicability has also been demonstrated in radar signal processing, including SAR imaging and through-the-wall radar imaging [26]. However, conventional RPCA relies on iterative singular value decomposition (SVD)-based thresholding operations, which result in high computational complexity and limit its direct use in real-time radar processing. To overcome this limitation, algorithm unfolding has been introduced to map each iteration of an iterative optimization algorithm to a corresponding neural network layer [27]. In particular, unfolded RPCA approaches have attracted attention because they preserve model-based interpretability while improving convergence speed and computational efficiency [28,29].

In this paper, we propose an unfolded RPCA network for efficient mitigation of inter-transmitter code interference in MIMO PMCW systems. The proposed network is built upon a neural architecture derived from the iterative update procedure of RPCA. By reflecting the low-rank interference and sparse target structure observed in the RD domain, and by introducing stage-wise learnable parameters together with Doppler-aware thresholding, the proposed network improves interference mitigation performance over existing methods. In addition, the proposed network adopts an efficient low-rank update that avoids explicit SVD, which enhances its potential for real-time implementation. The main contributions of this work are summarized as follows:

- We analyze the structural characteristics of inter-transmitter code interference in the RD domain of MIMO PMCW systems and formulate the interference mitigation problem as a low-rank and sparse decomposition problem.
- An unfolded RPCA network tailored for complex-valued RD domain data is proposed, in which the conventional SVD-based low-rank update is replaced with an efficient factorized SVD-free structure. The proposed architecture also incorporates stage-wise learnable optimization parameters for adaptive interference suppression.
- Simulations under various transmitter configurations demonstrate that the proposed method achieves interference mitigation performance comparable to conventional RPCA while providing lower latency than conventional RPCA and improved mitigation performance over deep learning-based baselines.

The remainder of this article is organized as follows. Section 2 presents the signal model of the PMCW systems and analyzes inter-transmitter code interference in MIMO PMCW systems. In Section 3, the interference mitigation problem based on RPCA is formulated and the proposed unfolded RPCA network architecture is described. The performance evaluation results through simulations are presented in Section 4. Finally, Section 5 concludes this article.

## 2. System Model

### 2.1. Range–Velocity Estimation in PMCW Systems

In this section, we describe the signal model of the PMCW system and the procedure for estimating the range and relative velocity of the target. The PMCW system transmits a continuous-wave signal whose carrier phase is modulated according to a pseudorandom binary sequence (PRBS). The PRBS consists of $N_{c}$ chips, each with duration $T_{c}$. Accordingly, the duration of one sequence is defined as $T_{s}=N_{c}T_{c}$. To perform a measurement, the same coded sequence is repeated $N_{s}$ times, and the transmitted signal modulated onto the carrier frequency $f_{c}$ can be expressed as

(1) $$s\left(t\right)=\sum_{n=0}^{N_{c}-1}\sum_{m=0}^{N_{s}-1}\exp\left\{j(2\pi f_{c}t+\phi_{n})\right\}\mathrm{rect}\left(\frac{t-mT_{s}-nT_{c}}{T_{c}}\right),$$

where *n* and *m* denote the chip index and sequence index, respectively. In addition, $\phi_{n}\in \left\{0,\;\pi \right\}$ represents the *n*-th chip phase determined by the PRBS and rect(·) denotes a rectangular pulse function.

The transmitted signal is reflected by $N_{t}$ targets and received at the receiver of the PMCW system. The received signal can be expressed as

(2) $$s_{\mathrm{Rx}}\left(t\right)=\sum_{i=1}^{N_{t}}\alpha_{i}s\left(t-\tau_{i}\right)\exp\left(-j2\pi f_{D,\;i}t\right),$$

where *i* denotes the index of the target and $\alpha_{i}$ denotes the complex reflection coefficient of the *i*-th target, which incorporates both path loss and radar cross-section. In addition, $\tau_{i}=\frac{2R_{i}}{c}$ and $f_{D,\;i}=\frac{2v_{i}f_{c}}{c}$ represent the round-trip propagation delay corresponding to the range $R_{i}$ of the *i*-th target and the Doppler frequency shift corresponding to the relative velocity of the target $v_{i}$, where *c* is the speed of light. The received signal is down-converted to baseband by mixing with the carrier signal and subsequently low-pass filtered. After analog-to-digital conversion, the signal is sampled at time instants $t=nT_{c}+mT_{s}$. The sampled signal can be expressed as

(3) $$s\left[n,\;m\right]=\sum_{i=1}^{N_{t}}\alpha_{i}c\left[n-n_{d,\;i}\right]\exp\left(-j2\pi f_{D,\;i}mT_{s}\right).$$

Here, c[n] denotes the value of the transmitted PRBS sampled at intervals of $T_{c}$ and $n_{d,\;i}=\left\lfloor \frac{\tau_{i}}{T_{c}}\right\rfloor$ represents the chip index shift corresponding to the propagation delay of the *i*-th target.

**Figure 1:**
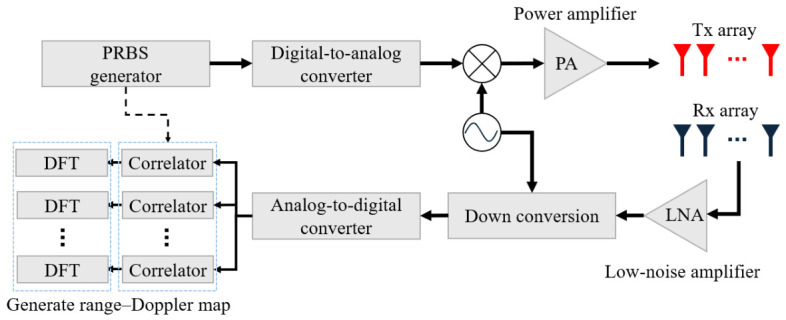
Signal-processing block diagram of the PMCW system.

To estimate the range information of the targets, pulse compression is performed along the fast-time axis by correlating the received signal with the transmitted PRBS. The correlation output can be expressed as

(4) $$r\left[p,\;m\right]=\sum_{n=0}^{N_{c}-1}s\left[n,\;m\right]c^{*}\left[\mathrm{mod}\left(n-p,\;N_{c}\right)\right],$$

where *p*, ${\left(\cdot \right)}^{*}$, and mod(·) denote the range-bin index, the complex conjugate, and the modulo operator, respectively. This operation exploits the autocorrelation property of the PRBS, which produces a peak at the delay index of each target. After range compression, a discrete Fourier transform is applied along the slow-time axis to estimate the relative velocities of the targets. The resulting RD response can be expressed as

(5) $$S\left[p,\;q\right]=\sum_{m=0}^{N_{s}-1}r\left[p,\;m\right]\exp\left(-j\frac{2\pi qm}{N_{s}}\right),$$

where *q* denotes the Doppler-bin index in the frequency domain. The complex-valued matrix S[p,q] represents the two-dimensional (2D) RD response of the radar system. The RD map is constructed from the power of the RD response, given by ${\left|S[p,\;q]\right|}^{2}$. The locations of the dominant peaks in the RD map provide estimates of the range $R_{i}$ and relative velocity $v_{i}$ of each target. In the subsequent sections, the complex-valued RD response matrix is used as the observation matrix for interference mitigation, whereas the RD map refers to its power representation used for visualization and detection. The overall signal-processing procedure is illustrated in Figure 1.

### 2.2. Inter-Transmitter Code Interference Analysis in MIMO PMCW Systems

**Figure 2:**
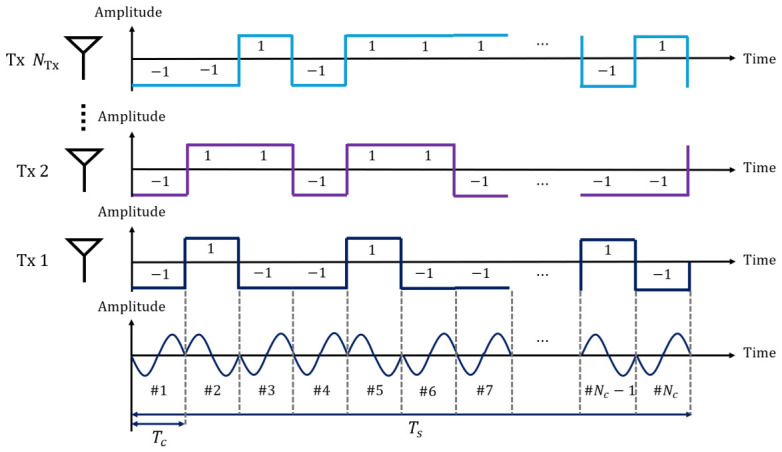
Illustration of fast-time CDM in a MIMO PMCW system.

In a MIMO PMCW system with $N_{\mathrm{Tx}}$ transmit antennas, fast-time CDM enables simultaneous transmission from all transmitters, with each assigned a distinct PRBS as shown in Figure 2. Hereafter, the transmitter-specific PRBS is also referred to as a code. As a result, the received signal at the *l*-th receiver is given by the superposition of target echoes associated with all transmitters and can be expressed as

(6) $$y_{l}\left[n,\;m\right]=\sum_{i=1}^{N_{t}}\sum_{k=1}^{N_{\mathrm{Tx}}}\alpha_{i}\;c_{k}\left[n-n_{d,\;i}\right]\;\psi_{i,\;k,\;l}\left[m\right]+w_{l}\left[n,\;m\right],$$

where *k*, $c_{k}\left[n\right]$, and $w_{l}$ denote the index of the transmitter, the code assigned to the *k*-th transmitter, and the additive noise at the *l*-th receiver, respectively. In addition, ψ represents the phase term that accounts for both the Doppler-induced phase progression and the array-dependent phase offset determined by the uniform linear array configuration of the MIMO system. To extract the signal component corresponding to each transmitter, a demultiplexing operation is performed. This is achieved by correlating the received signal with the code assigned to the desired transmitter using a matched filter, and can be expressed as

(7) $$r_{k,\;l}\left[p,\;m\right]=\sum_{n=0}^{N_{c}-1}y_{l}\left[n,\;m\right]c_{k}^{*}\left[\mathrm{mod}\left(n-p,\;N_{c}\right)\right],$$

which follows the same procedure for range estimation described in the previous section. In the ideal case, only the reflected signal component corresponding to the desired transmitter remains in the correlation output, and the contributions from all other transmitters are fully suppressed.

However, due to the simultaneous occupation of the same time and frequency resources by multiple transmitters in fast-time CDM MIMO systems, undesired cross-correlation components from the codes assigned to the other transmitters remain in the matched-filter output. The correlation result in (7) can be decomposed into the autocorrelation term and cross-correlation terms as

(8) $$\begin{array}{cc} r_{k,\;l}\left[p,\;m\right] & =\sum_{i=1}^{N_{t}}\sum_{j=1}^{N_{\mathrm{Tx}}}\alpha_{i}\;\psi_{i,\;j,\;l}\left[m\right]\sum_{n=0}^{N_{c}-1}c_{j}\left[n-n_{d,\;i}\right]\;c_{k}^{*}\left[\mathrm{mod}(n-p,N_{c})\right]\\ \; & =\sum_{i=1}^{N_{t}}\alpha_{i}\;\psi_{i,\;k,\;l}\left[m\right]\sum_{n=0}^{N_{c}-1}c_{k}\left[n-n_{d,\;i}\right]\;c_{k}^{*}\left[\mathrm{mod}(n-p,N_{c})\right]\\ \; & \;+\sum_{i=1}^{N_{t}}\sum_{\begin{array}{c} j=1\\ j\neq k \end{array}}^{N_{\mathrm{Tx}}}\alpha_{i}\;\psi_{i,\;j,\;l}\left[m\right]\sum_{n=0}^{N_{c}-1}c_{j}\left[n-n_{d,\;i}\right]\;c_{k}^{*}\left[\mathrm{mod}(n-p,N_{c})\right]. \end{array}$$

**Figure 3:**
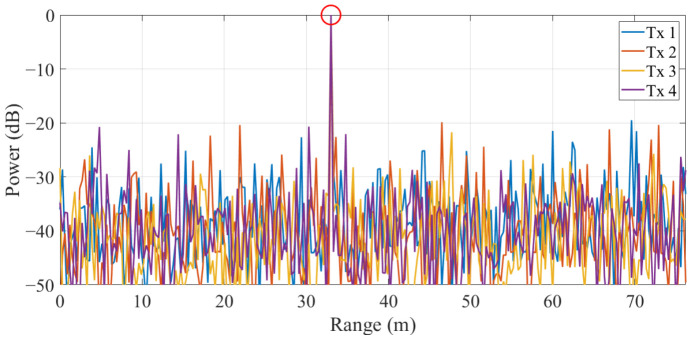
Example of a range profile after transmitter-wise matched filtering in the MIMO PMCW system with $N_{\mathrm{Tx}}=4$, distinct *m*-sequences assigned to the transmitters, a target at 33 m, and SNR = 10 dB. The red circle indicates the target peak.

Here, the first term corresponding to j=k represents the desired response obtained from the autocorrelation of the code assigned to the *k*-th transmitter. This term achieves its peak value when the range-bin index matches the delay index $n_{d,\;i}$, which corresponds to the round-trip propagation delay of the *i*-th target. In contrast, the second term corresponding to j≠k corresponds to inter-transmitter code interference caused by the cross-correlation with the codes assigned to the other transmitters. Although these cross-correlation terms are ideally minimized through code design, complete suppression is not achieved in practice. Finite code length and Doppler-induced mismatch reduce the effective orthogonality among the assigned codes, and additive noise further contaminates the correlation output. As a result, residual components from other transmitters may remain after the correlation process, leading to interference in the correlation output. Figure 3 shows the transmitter-wise range profiles, where interference components originating from other transmitters are observed in addition to the peak corresponding to the target.

**Figure 4:**
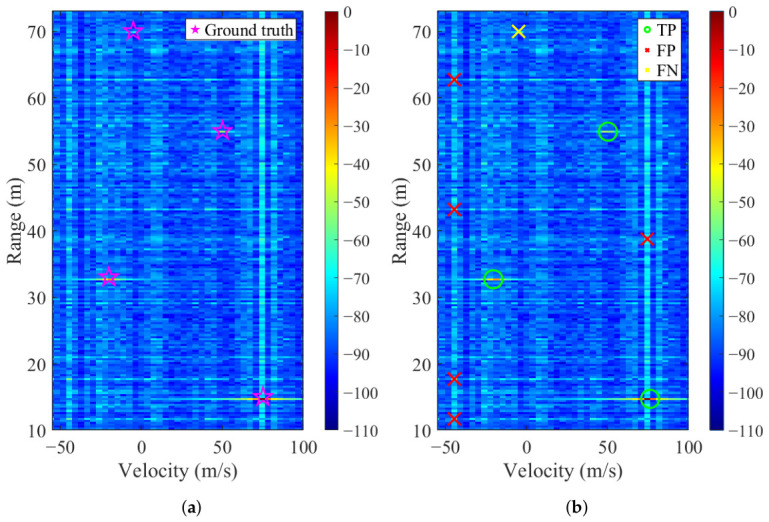
Effect of inter-transmitter code interference on target detection in the RD domain for a MIMO PMCW scenario with $N_{\mathrm{Tx}}=4$, distinct *m*-sequences assigned to the transmitters, SNR = 10 dB, and four targets located at (33 m, −20 m/s), (15 m, 75 m/s), (70 m, −5 m/s), and (55 m, 50 m/s). (**a**) RD map corrupted by structured inter-transmitter interference. (**b**) CFAR detection results, where true positive (TP), false positive (FP), and false negative (FN) denote a correctly detected target, an incorrect alarm at a non-target, and a missed detection, respectively.

These residual interference components can lead to erroneous target detections in the RD map. As shown in Figure 4a, the inter-transmitter interference manifests as a structured background component that spreads broadly across the RD domain. This interference forms spatially correlated patterns that elevate the background level over a wide region, whereas target echoes remain localized and occupy only a small number of RD cells. From this perspective, the interference can be interpreted as a low-rank component with strong spatial structure, and the target echoes can be regarded as sparse components. When constant false alarm rate (CFAR) detection is applied to the RD map, as shown in Figure 4b, the structured interference distorts the local background estimation and causes both false alarms and missed detections. In particular, interference-dominant regions may be falsely declared as targets, whereas targets with low signal power may be masked by the elevated background level and remain undetected. These observations motivate the use of an RPCA-based formulation for interference mitigation, in which the observed complex-valued RD response is decomposed into a low-rank interference component and a sparse target component.

## 3. Proposed Methods for Inter-Transmitter Code Interference Mitigation

### 3.1. Problem Formulation

RPCA addresses the problem of decomposing an input matrix *D* into the sum of a low-rank component *L* and a sparse component *S*. In this work, the input observation matrix *D* corresponds to the complex-valued RD response matrix obtained after range compression and relative velocity estimation. The decomposition is typically formulated as the following convex optimization problem, which minimizes the nuclear norm of *L* and the element-wise $\ell_{1}$ norm of *S*:

(9) $$\min_{L,\;S}{\;∥L∥}_{*}+\lambda {∥S∥}_{1}\;\mathrm{subject}\;\mathrm{to}\;D=L+S,$$

where ${∥\cdot ∥}_{*}$ and ${∥\cdot ∥}_{1}$ denote the nuclear norm and the element-wise $\ell_{1}$-norm, respectively, and λ is a regularization parameter that controls the trade-off between low-rankness and sparsity. To solve this optimization problem, the inexact augmented Lagrangian method (IALM) is employed, which reformulates the problem using the following augmented Lagrangian:

(10) $$L\left(L,\;S,\;Y,\;\mu \right)={∥L∥}_{*}+{\lambda ∥S∥}_{1}+\langle Y,\;D-L-S\rangle +\frac{\mu }{2}{∥D-L-S∥}_{F}^{2},$$

where 〈A,B〉, ${∥\cdot ∥}_{F}$, *Y*, and μ denote the Frobenius inner product, the Frobenius norm, the dual variable (i.e., Lagrange multiplier), and the penalty parameter that controls the strength of constraint enforcement, respectively. The optimization proceeds via alternating minimization, and each iteration consists of the following four updates:

- *L*-update: $L^{\left(u+1\right)}=\arg\min_{L}\;L\left(L,\;S^{\left(u\right)},\;Y^{\left(u\right)},\;\mu^{\left(u\right)}\right)$;
- *S*-update: $S^{\left(u+1\right)}=\arg\min_{S}\;L\left(L^{\left(u+1\right)},\;S,\;Y^{\left(u\right)},\;\mu^{\left(u\right)}\right)$;
- Dual variable update: $Y^{\left(u+1\right)}=Y^{\left(u\right)}+\mu^{\left(u\right)}\left(D-L^{\left(u+1\right)}-S^{\left(u+1\right)}\right)$;
- Penalty parameter update: $\mu^{\left(u+1\right)}=\rho \mu^{\left(u\right)}$.

Here *u* and ρ denote the iteration index and the penalty growth factor that accelerates convergence by gradually increasing μ, respectively. The *L*-update is performed by applying singular value thresholding (SVT) to the matrix $Q^{\left(u\right)}=D-S^{\left(u\right)}+Y^{\left(u\right)}/\mu^{\left(u\right)}$. Specifically, the SVD of $Q^{\left(u\right)}$ is computed as $Q^{\left(u\right)}=U\Sigma V^{H}$, and SVT is then applied using the operator $\Theta_{1/\mu^{\left(u\right)}}\left(\cdot \right)$ to obtain $L^{\left(u+1\right)}=U\Theta_{1/\mu^{\left(u\right)}}\left(\Sigma \right)V^{H}$. The *S*-update is computed element-wise using the $\ell_{1}$ soft-thresholding operator. By iteratively repeating these updates, the decomposition satisfying D=L+S is obtained.

However, the SVD operation required in the *L*-update step incurs high computational complexity. For an n×m matrix, SVD has a complexity of O(nmmin(n,m)), which limits computational efficiency and introduces significant latency. In applications involving large-scale or high-resolution radar data, this step becomes a major computational bottleneck in the RPCA-based interference mitigation pipeline. To address this limitation, we propose an unfolded RPCA network in which the SVD-based low-rank update is replaced with an SVD-free factorized structure. The proposed network is directly applicable to complex-valued 2D data matrices in the RD domain and is designed to provide effective interference mitigation with low computational latency.

### 3.2. Proposed Unfolded RPCA Network

The proposed unfolded RPCA network maps the iterative structure of the IALM-based RPCA algorithm into a deep network architecture. Each iteration, consisting of the updates for *L*, *S*, and *Y*, is implemented as a network stage, and the stages are connected sequentially to form a trainable feed-forward architecture. While the conventional IALM framework relies on SVD in every *L*-update step, the proposed network replaces the SVD-based low-rank update with a factorized representation of *L* using the product of two low-dimensional matrices. This factorization allows the network to estimate *L* through efficient linear algebra operations instead of computationally expensive SVD operations. The entire network consists of $N_{k}$ stages, where each stage corresponds to a single IALM iteration.

#### 3.2.1. SVD-Free Factorized *L*-Update

In the conventional IALM framework, the *L*-update relies on SVT, which requires a full SVD at every iteration. For an n×m matrix, this incurs a complexity of O(nmmin(n,m)), which becomes a major computational bottleneck when the update is repeated across multiple unfolding stages. To eliminate this bottleneck, the proposed network approximates *L* as the product of two lower-dimensional factor matrices, as given by

(11) $$L\approx UV^{H},$$

where $U\in C^{n\times r}$ and $V\in C^{m\times r}$ are the left and right factor matrices that collectively encode the low-rank component, and *r* denotes the factorization rank satisfying r≪min(n,m). This formulation completely avoids the computation of singular values and singular vectors, and estimates *L* using only simple linear algebraic operations. Given the auxiliary matrix *Q* defined in Section 3.1, *U* and *V* are estimated by minimization of the regularized least-squares objective, which can be expressed as

(12) $$\min_{U,\;V}{∥Q-UV^{H}∥}_{F}^{2}+\frac{1}{\mu }\left({∥U∥}_{F}^{2}+{∥V∥}_{F}^{2}\right).$$

Since (12) is non-convex jointly in (U,V), the alternating least squares (ALS) algorithm is applied to solve the problem [30]. Each subproblem is a ridge regression problem admitting a closed-form solution, which makes each individual factor update well-defined. Applying the optimality condition to each subproblem in turn, the factor matrices are updated as

(13) $$\begin{array}{c} U_{\mathrm{new}}=QV_{\mathrm{old}}{\left(V_{\mathrm{old}}^{H}V_{\mathrm{old}}+\frac{1}{\mu }I\right)}^{-1}, \end{array}$$

(14) $$\begin{array}{c} V_{\mathrm{new}}=Q^{H}U_{\mathrm{new}}{\left(U_{\mathrm{new}}^{H}U_{\mathrm{new}}+\frac{1}{\mu }I\right)}^{-1}. \end{array}$$

This approach requires O(nmr) computational complexity per ALS iteration, which is lower than the O(nmmin(n,m)) complexity of SVD. In practice, the number of ALS iterations per stage is limited to 1–3. Within the unfolding framework, a small number of ALS passes per stage already provide a sufficiently accurate low-rank approximation, and additional iterations offer only marginal improvement. A fully converged low-rank estimate is not required at each stage because the stage-wise learnable parameters are optimized together with the factorized update during end-to-end training. The factorization rank *r* is set as a fixed hyperparameter satisfying r≪min(n,m). Specifically, *r* is chosen large enough to capture the low-rank structure in the complex-valued RD response, without incurring excessive per-stage latency. This design reduces computational complexity while preserving sufficient approximation accuracy for interference mitigation. This factorized *L*-update method is integrated into each stage of the proposed unfolding network, as described in the following subsection.

#### 3.2.2. Deep Unfolding Architecture

**Figure 5:**
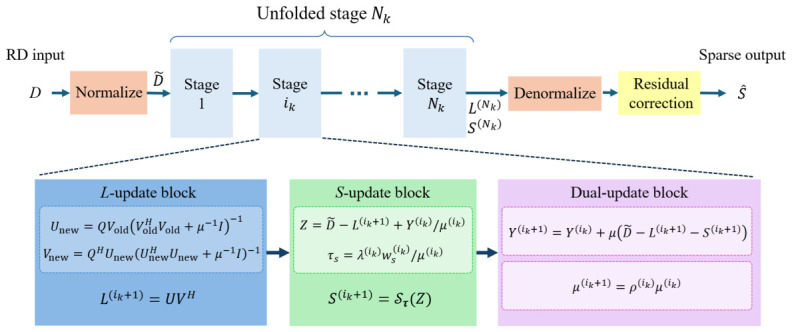
Architecture of the proposed unfolded RPCA network for inter-transmitter code interference mitigation.

The proposed network unfolds the IALM algorithm into a fixed-depth feed-forward architecture consisting of $N_{k}$ sequential stages, as shown in Figure 5. Each stage corresponds to one IALM iteration and operates by passing the updated tuple $\left(L^{\left(i_{k}\right)},\;S^{\left(i_{k}\right)},\;Y^{\left(i_{k}\right)},\;\mu^{\left(i_{k}\right)}\right)$ to the next stage. Unlike conventional IALM in which the key update parameters are fixed scalars, the proposed network assigns independent learnable parameters to each stage $i_{k}$ and additionally incorporates a globally shared residual correction parameter. This design enables the network to adaptively control the sparsity threshold, Doppler-aware threshold profile, and penalty growth rate at each stage. In addition, the residual correction coefficient is shared across all stages and optimized globally during training.

##### Input Normalization

Prior to entering the first stage, the input matrix $D\in C^{n\times m}$ is normalized on a per-sample basis by its Frobenius norm, given by $\tilde{D}=D/{∥D∥}_{F}$. This normalization ensures that the optimization operates on a consistent scale regardless of the absolute power of the input, which stabilizes the training process across all $N_{k}$ stages. The normalization factor ${∥D∥}_{F}$ is stored and applied inversely to the network output to recover the correct scale.

##### Stage Structure

Within stage $i_{k}$, the three sub-steps are executed sequentially as follows:

*(i) L*-update: The auxiliary matrix $Q^{\left(i_{k}\right)}$ is formed from $\tilde{D}$, $S^{\left(i_{k}\right)}$, $Y^{\left(i_{k}\right)}$, and $\mu^{\left(i_{k}\right)}$. The low-rank factors $U^{\left(i_{k}+1\right)}$ and $V^{\left(i_{k}+1\right)}$ are then updated via the ALS procedure described in Section 3.2.1, and the low-rank estimate is reconstructed as $L^{\left(i_{k}+1\right)}=U^{\left(i_{k}+1\right)}{\left(V^{\left(i_{k}+1\right)}\right)}^{H}$.

*(ii) S*-update: The sparse component is updated by applying complex soft-thresholding, and is given by

(15) $$S^{\left(i_{k}+1\right)}=S_{\tau_{q}^{\left(i_{k}\right)}}\left(\tilde{D}-L^{\left(i_{k}+1\right)}+Y^{\left(i_{k}\right)}/\mu^{\left(i_{k}\right)}\right),$$

where $S_{\tau }\left(z\right)=\max\left(|z|-\tau ,0\right)\times \frac{z}{\left|z\right|}$ denotes the soft-thresholding operator applied element-wise, and $\tau_{q}^{\left(i_{k}\right)}=\lambda^{\left(i_{k}\right)}w_{q}^{\left(i_{k}\right)}/\mu^{\left(i_{k}\right)}$ denotes the threshold defined per Doppler-bin index *q*. Here, the learnable weight $w_{q}^{\left(i_{k}\right)}=\mathrm{softplus}\left({\tilde{w}}_{q}^{\left(i_{k}\right)}\right)$ enables the network to assign stronger thresholding at interference-dominant bins and weaker thresholding at target-dominant bins. The per-stage sparsity parameter $\lambda^{\left(i_{k}\right)}$ is obtained as $\lambda^{\left(i_{k}\right)}=\exp\left({\tilde{\lambda }}^{\left(i_{k}\right)}\right)$, where ${\tilde{\lambda }}^{\left(i_{k}\right)}$ is an unconstrained learnable scalar. This exponential reparameterization guarantees $\lambda^{\left(i_{k}\right)}>0$ throughout training without requiring explicit constraints on the parameter.

*(iii)* Dual variable and penalty update: The dual variable is updated as

(16) $$Y^{\left(i_{k}+1\right)}=Y^{\left(i_{k}\right)}+\mu^{\left(i_{k}\right)}\left(\tilde{D}-L^{\left(i_{k}+1\right)}-S^{\left(i_{k}+1\right)}\right).$$

In addition, the penalty parameter is then updated as

(17) $$\mu^{\left(i_{k}+1\right)}=\rho^{\left(i_{k}\right)}\mu^{\left(i_{k}\right)},$$

where $\rho^{\left(i_{k}\right)}$ denotes a learnable stage-wise growth rate. To ensure $\rho^{\left(i_{k}\right)}>1$, it is parameterized using an unconstrained scalar ${\tilde{\rho }}^{\left(i_{k}\right)}$ through a softplus-based transformation. As a result, $\mu^{\left(i_{k}\right)}$ increases monotonically across stages. Such a monotonic increase is consistent with the convergence behavior typically assumed in IALM and is guaranteed to hold throughout gradient-based optimization without requiring explicit constraints. Furthermore, by assigning an independent $\rho^{\left(i_{k}\right)}$ to each stage, the proposed network can learn a stage-wise penalty schedule, which offers greater flexibility than the conventional IALM that applies a single fixed scalar ρ uniformly across all iterations.

##### Output and Loss Function

The final sparse estimate $\hat{S}$ is obtained in two steps. First, the last-stage outputs are denormalized as $\tilde{S}=S^{\left(N_{k}\right)}{∥D∥}_{F}$ and $\tilde{L}=L^{\left(N_{k}\right)}{∥D∥}_{F}$. The final output is then obtained through residual correction and can be expressed as

(18) $$\hat{S}=\left(1-\delta \right)\tilde{S}+\delta \left(D-\tilde{L}\right),$$

where δ denotes a globally shared learnable parameter that controls the relative contribution of the two estimates, and is jointly optimized during training. The network is trained end-to-end by minimizing the following complex mean squared error (MSE) loss over labeled training pairs $\left(D,\;S_{\mathrm{gt}}\right)$, which can be expressed as

(19) Lloss=∥Re(S^)−Re(Sgt)∥F2+∥Im(S^)−Im(Sgt)∥F2,

where $S_{\mathrm{gt}}$ denotes the ground-truth sparse component, and Re(·) and Im(·) denote the real and imaginary parts, respectively. The learnable parameters of the network are defined as $P={\left\{{\tilde{\lambda }}^{\left(i_{k}\right)},\;{\tilde{\rho }}^{\left(i_{k}\right)},\;{\tilde{w}}_{q}^{\left(i_{k}\right)}\right\}}_{i_{k}=1}^{N_{k}}$ along with the globally shared residual correction parameter δ. All parameters are jointly optimized via the Adam optimizer with gradient clipping and a cosine annealing learning rate schedule.

## 4. Simulation Results

### 4.1. Simulation Setup and Training Configuration

Simulations were conducted for a MIMO PMCW system operating at 77 GHz. Each transmitter used an *m*-sequence-based code of length 255 with a chip duration of 2 ns, which corresponds to a signal bandwidth of 500 MHz. The primary system parameters are summarized in Table 1.

The training dataset was generated under transmitter configurations with $N_{\mathrm{Tx}}\in${2,3,…,12}. For each configuration, 200 independent scenes were simulated, resulting in a total of 2200 samples. Here, 90% of the samples were used for training and the remaining 10% were used for validation. The split was performed independently for each configuration to maintain a balanced distribution across all $N_{\mathrm{Tx}}$ cases. For each scene, the polynomial indices defining the transmitter-specific *m*-sequences were randomly selected from the code family [31]. The resulting inter-transmitter code interference appeared in the RD map and was modeled as the low-rank interference component *L*. Each scene was generated with a randomized number of targets (e.g., one to four), range (e.g., 10 m to 70 m), and relative velocity (e.g., −50 m/s to 50 m/s). The signal-to-noise ratio (SNR) of each sample was drawn uniformly from 0 to 20 dB. The ground-truth sparse component $S_{\mathrm{gt}}$ was obtained by applying the IALM algorithm to the complex-valued RD response *D* in the RD domain. Because the IALM algorithm converges to a solution of the underlying convex RPCA formulation, the resulting sparse component was used as the surrogate ground truth.

**Figure 6:**
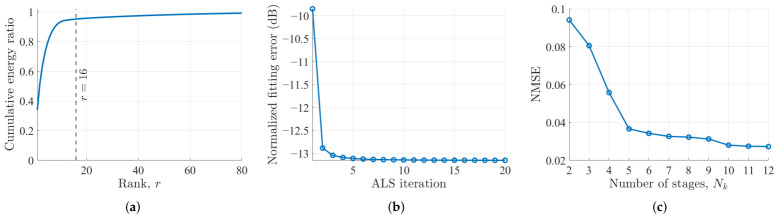
Hyperparameter selection analysis for the proposed network. (**a**) Cumulative energy ratio versus factorization rank *r*, (**b**) normalized low-rank fitting error versus the number of ALS iterations, and (**c**) validation NMSE versus the number of unfolding stages $N_{k}$.

The proposed network was implemented in PyTorch 2.11.0 and trained using the Adam optimizer with gradient clipping. The initial learning rate was set to $2\times 10^{-3}$ and followed a cosine annealing schedule with a minimum learning rate of $2\times 10^{-5}$. The batch size and the number of training epochs were set to 16 and 100, respectively. The residual correction coefficient δ was initialized at 0.5 and jointly optimized during training. The factorization rank *r* was set to 16 based on the cumulative singular value energy analysis of the estimated low-rank component. As shown in Figure 6a, the first 16 singular components captured more than 95% of the total energy on average. This result indicates that r=16 provides a reasonable approximation of the dominant low-rank structure while avoiding unnecessary computational and parameter overhead. The number of ALS iterations per stage was determined from the normalized low-rank fitting error of the factorized *L*-update. As shown in Figure 6b, the fitting error decreased rapidly during the first few iterations and showed only marginal improvement after two iterations. Thus, the number of ALS iterations per stage was set to two as a practical trade-off between fitting accuracy and computational cost. Since the factorized low-rank update is non-convex with respect to the two factor matrices, this analysis is intended to assess the empirical stability of the ALS update rather than to establish global optimality. The number of unfolding stages was selected based on the validation normalized MSE (NMSE) after training models with $N_{k}\in \left\{2,\;3,\;\ldots ,\;12\right\}$. This NMSE was computed using the sparse component reconstruction loss defined in (19), which measures the relative reconstruction error in the complex domain. As shown in Figure 6c, the validation NMSE decreased as $N_{k}$ increased and became nearly saturated after $N_{k}=10$. Therefore, $N_{k}=10$ was selected as a practical trade-off between sparse component reconstruction accuracy and computational cost.

### 4.2. Qualitative Analysis of Interference Mitigation Results

To evaluate the effectiveness of the proposed method, we compare it with three baseline approaches: conventional RPCA [22], learned iterative shrinkage–thresholding algorithm (LISTA) [32], and RPCANet [33]. Conventional RPCA decomposes the observation matrix into a low-rank component and a sparse component by solving a convex optimization problem, as described in Section 3.1. Although this approach achieves accurate separation by iteratively solving the optimization problem via IALM, it incurs high computational complexity due to the SVD required at every iteration. LISTA unfolds the iterative structure of ISTA into a finite-depth neural network under the algorithm unfolding framework. By replacing iterative optimization with learnable parameters, LISTA improves both convergence speed and recovery performance for sparse signal reconstruction. LISTA was selected as a baseline because it shares the algorithm unfolding perspective of the proposed method and provides a reference for evaluating sparse target component estimation. RPCANet is a deep unfolding network based on iterative RPCA. By unfolding convolutional neural network (CNN)-based optimization iterations into network layers, RPCANet accelerates convergence toward the low-rank and sparse solution while preserving the explicit decomposition structure of the RPCA formulation. Since RPCANet adopts the same RPCA unfolding framework as the proposed method, we compare the interference mitigation performance based on differences in network design strategies.

**Figure 7:**
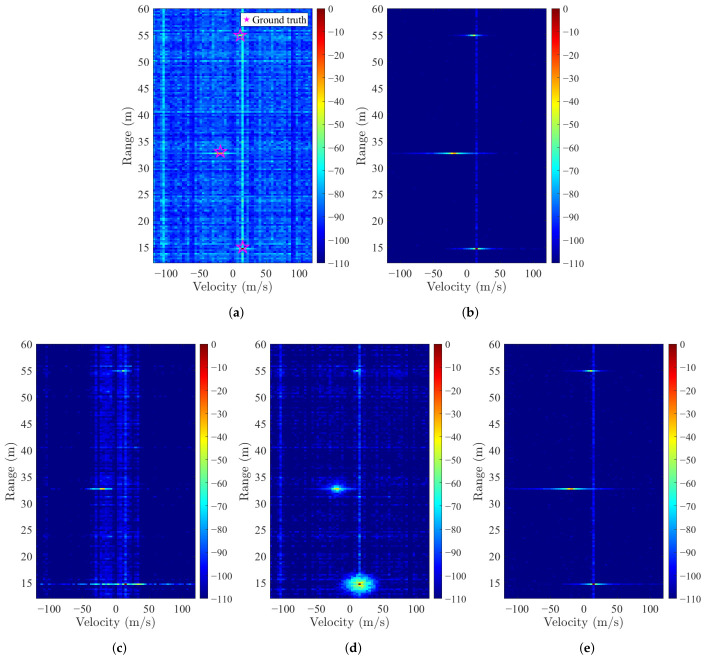
Qualitative comparison of RD domain interference mitigation results for the simulation scenario. (**a**) Interference-corrupted RD map. (**b**–**e**) RD maps reconstructed from the sparse components estimated by each method: (**b**) conventional RPCA, (**c**) LISTA, (**d**) RPCANet, and (**e**) the proposed method. All color scales are normalized to the range from −110 to 0 dB for fair comparison.

Figure 7 presents the simulation results obtained by different approaches for qualitative comparison. In this scenario, three targets were located at (15 m, 15 m/s), (33 m, −20 m/s), and (55 m, 10 m/s) in range and relative velocity, respectively, with an SNR of 10 dB. Figure 7a shows the RD map containing inter-transmitter code interference when $N_{\mathrm{Tx}}=4$. The interference manifested as strongly correlated components along the Doppler axis in the RD map, which significantly elevated the interference floor and obscured detection of the target echoes. As shown in Figure 7b, applying conventional RPCA substantially reduced the interference floor and rendered the peaks corresponding to the targets clearly distinguishable. This result indicates that conventional RPCA can achieve accurate interference mitigation at the expense of high computational cost. The result of applying LISTA is shown in Figure 7c. Since LISTA was designed solely for sparse signal recovery without a low-rank and sparse decomposition structure, it detected sparse target components but failed to effectively separate the interference components that exhibited low-rank structure, leaving residual background components in the output. Figure 7d shows the result of applying RPCANet. The finite receptive field of the CNN layers led to incorrect reconstruction of target-like components in the vicinity of the peaks corresponding to the targets and produced spreading artifacts around them. As a result, while target components were detected, the contrast between the target and background remained low, and the interference mitigation performance was inferior to that of the proposed method. The proposed method effectively suppressed interference while preserving sharp peaks corresponding to the targets. As shown in Figure 7e, the interference pattern observed in the interference-corrupted RD map was substantially reduced, and target echoes appeared as well-localized peaks. These results demonstrate that the proposed network effectively separated the low-rank interference and sparse target components.

**Figure 8:**
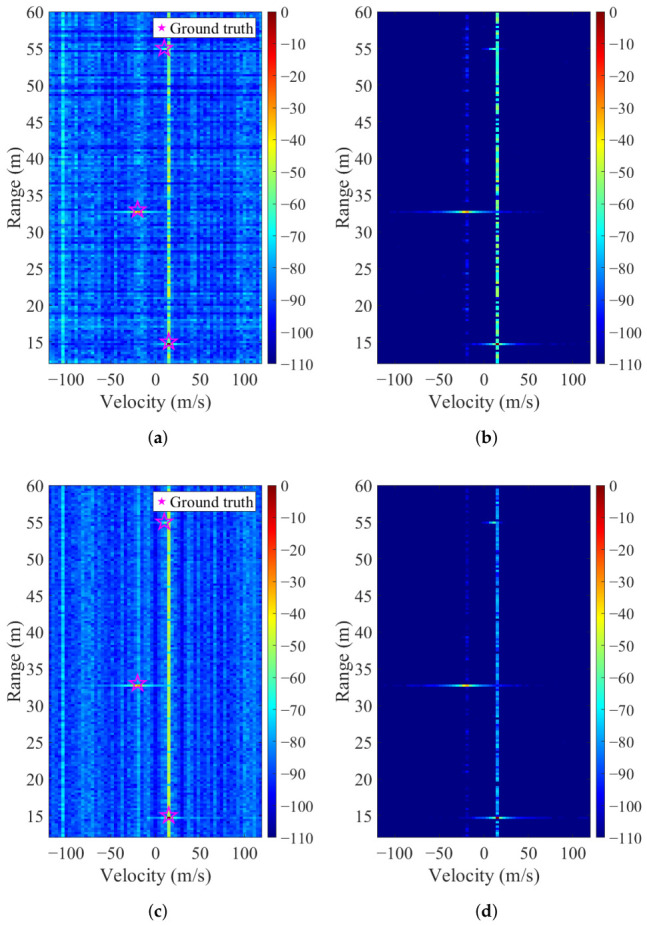
RD map comparison for different code families. (**a**) Interference-corrupted RD map using Gold codes, (**b**) sparse component RD map reconstructed by the proposed method using Gold codes, (**c**) interference-corrupted RD map using Kasami codes, and (**d**) sparse component RD map reconstructed by the proposed method using Kasami codes.

To further examine the generality of the proposed method, additional qualitative evaluations were conducted using Gold and Kasami codes as alternative code families. Although the main experiments employ *m*-sequences due to their favorable autocorrelation properties and simple generation, the proposed unfolded RPCA framework is not inherently tied to a specific code family. As shown in Figure 8, the proposed method effectively suppresses the structured interference and recovers well-localized target peaks for both Gold and Kasami codes. This result indicates that the proposed method is not limited to the *m*-sequence setting. The absolute level of residual interference may vary depending on the cross-correlation properties of the selected codes. Nevertheless, the observed RD maps indicate that the interference remains sufficiently separable from the sparse target component in the tested code families, which supports the broader applicability of the proposed approach.

### 4.3. Quantitative Performance Evaluation

In this section, quantitative performance is evaluated using the interference-mitigated RD maps obtained by each method. To assess the RD-map quality related to target detectability following interference mitigation, two metrics are adopted: the peak-to-sidelobe ratio (PSR) and the integrated sidelobe level ratio (ISLR). The PSR and ISLR values are computed from the RD maps reconstructed from the estimated sparse components. Both metrics are adapted to quantify the RD-map quality related to target detectability, as given by

(20) $$\begin{array}{cc} \mathrm{PSR} & =10\log_{10}\frac{\frac{1}{\left|M\right|}\sum_{(p,\;q)\in M}{\left|S\left[p,\;q\right]\right|}^{2}}{\frac{1}{\left|S\right|}\sum_{(p,\;q)\in S}{\left|S\left[p,\;q\right]\right|}^{2}}, \end{array}$$

(21) $$\begin{array}{cc} \mathrm{ISLR} & =10\log_{10}\frac{\sum_{(p,\;q)\in S}{\left|S\left[p,\;q\right]\right|}^{2}}{\sum_{(p,\;q)\in M}{\left|S\left[p,\;q\right]\right|}^{2}}, \end{array}$$

where M and S denote the mainlobe region defined within ±1 cells from the target along both the range and Doppler axes, and the sidelobe region defined as the non-mainlobe region after excluding the guard cells, respectively. Unlike the conventional PSR, which is based on the peak sidelobe at a single cell, the PSR used in this work employs the mean power over the sidelobe region S as the reference in order to reflect the overall level of distributed residual interference in the non-mainlobe region of the RD domain. A higher PSR value indicates that the peak corresponding to the target is more prominent relative to the surrounding interference and sidelobe levels. The ISLR is defined as the ratio of the total energy distributed over the sidelobe region S to the energy concentrated within the mainlobe region M. While PSR reflects the per-cell power contrast between the mainlobe and sidelobe regions, ISLR captures the degree to which the total signal energy is concentrated within the mainlobe. A lower ISLR value indicates that the target energy is well concentrated within the mainlobe and that residual interference has been effectively suppressed.

**Figure 9:**
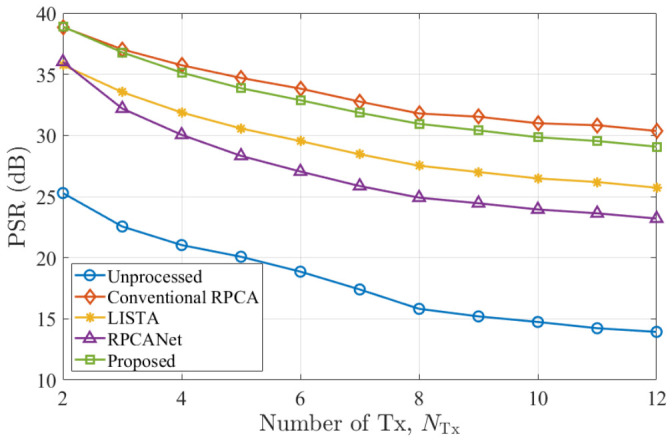
PSR measured from the reconstructed RD maps versus the number of transmitters for different interference mitigation methods.

**Figure 10:**
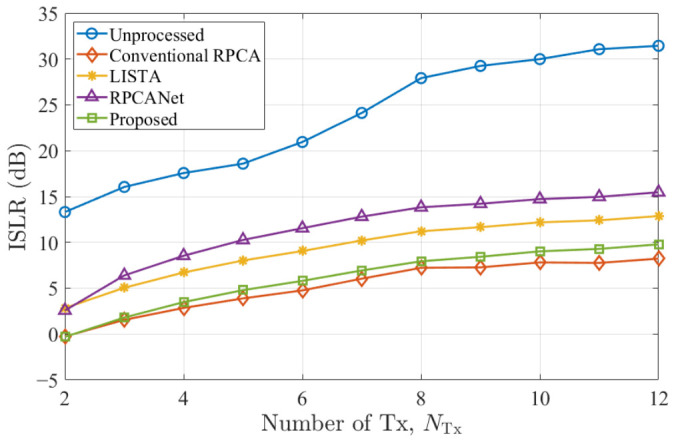
ISLR measured from the reconstructed RD maps versus the number of transmitters for different interference mitigation methods.

Because the level of inter-transmitter code interference depends on the number of simultaneously active transmitters, PSR and ISLR were evaluated as functions of $N_{\mathrm{Tx}}$. Figure 9 shows the PSR results for different numbers of transmitters. Conventional RPCA achieved an average PSR improvement of approximately 15.39 dB over the unprocessed case through SVD-based low-rank separation. LISTA yielded an average improvement of 11.23 dB through recovery of the sparse target component. On the other hand, RPCANet achieved a PSR that was 6.26 dB lower than that of conventional RPCA, which was attributed to residual sidelobes that were broadly distributed around the peaks corresponding to the targets. The proposed method achieved an average PSR improvement of 14.56 dB over the unprocessed case, which was comparable to that of conventional RPCA. The ISLR as a function of the number of transmitters is illustrated in Figure 10. Conventional RPCA achieved the lowest ISLR across all transmitter configurations, with an average improvement of 18.46 dB over the unprocessed case. LISTA and RPCANet reduced the ISLR by an average of 14.36 dB and 12.26 dB, respectively, although their interference mitigation performance remained limited relative to conventional RPCA. The proposed method exhibited an ISLR that was on average 0.89 dB higher than that of conventional RPCA. Nevertheless, this difference is acceptable when the inference latency reported below is taken into account, and the proposed method still provides practical interference mitigation performance.

To evaluate the computational efficiency of each method, the inference latency was measured and is summarized in Table 2. All measurements were conducted on an NVIDIA GeForce RTX 5070 Ti graphics processing unit equipped with 16 GB of GDDR7 memory [34]. Conventional RPCA effectively separated interference through SVD-based low-rank updates. However, its high computational complexity resulted in a latency of 526.712 ms, which failed to meet the real-time processing requirements of MIMO PMCW systems. LISTA achieved the lowest latency of 15.215 ms through independent vector operations, and RPCANet achieved a latency of 53.876 ms by virtue of its CNN-based unfolded RPCA architecture. Although both LISTA and RPCANet reduced latency through their unfolded structures and efficient model designs, their interference mitigation performance remained limited relative to conventional RPCA. The proposed network replaced the SVD operation with a factorized decomposition to reduce computational complexity and achieved a latency of 24.812 ms, which was 21.2 times lower than that of conventional RPCA. These results indicate that the proposed method achieves both effective mitigation of inter-transmitter code interference and computational efficiency favorable for real-time processing in MIMO PMCW systems.

## 5. Conclusions

In this paper, we proposed an unfolded RPCA network for efficient mitigation of inter-transmitter code interference in MIMO PMCW systems. We showed that the inter-transmitter code interference exhibited a low-rank structure in the RD domain, whereas target echoes retained sparse characteristics, which motivated the application of RPCA to the interference mitigation problem. The proposed network unfolded the IALM-based RPCA algorithm into a fixed-depth feed-forward architecture, in which the SVD operation was replaced by a factorized low-rank approximation solved via ALS to reduce computational complexity. Simulation results demonstrated that the proposed method improved PSR and ISLR by 14.56 dB and 17.57 dB over the unprocessed case, respectively, while achieving interference mitigation performance comparable to conventional RPCA and reducing inference latency by a factor of 21.2. Compared with the unfolding-based baselines such as LISTA and RPCANet, the proposed method achieved superior interference mitigation with competitive computational efficiency. These results confirm that the proposed method is a practical solution for real-time interference mitigation in MIMO PMCW systems.

## Figures and Tables

**Table 1:** MIMO PMCW system parameters used in simulations.

Parameter	Value
Carrier frequency, $f_{c}$	77 GHz
Chip duration, $T_{c}$	2 ns
Length of PRBS, $N_{c}$	255
Number of sequences, $N_{s}$	256
Range resolution	0.3 m
Maximum detectable range	76.5 m
Sequence type	*m*-sequence

**Table 2:** Comparison of inference latency for different interference mitigation methods.

Methods	Latency
Conventional RPCA	526.712 ms
LISTA	15.215 ms
RPCANet	53.876 ms
Proposed	24.812 ms

## Data Availability

The data presented in this study are available on request from the corresponding author. The data are not publicly available due to privacy.

## Abbreviations

The following abbreviations are used in this manuscript:

ALS	Alternating least squares
CDM	Code-division multiplexing
CFAR	Constant false alarm rate
CNN	Convolutional neural network
FMCW	Frequency-modulated continuous wave
IALM	Inexact augmented Lagrangian method
ISAC	Integrated sensing and communication
ISLR	Integrated sidelobe level ratio
LISTA	Learned iterative shrinkage–thresholding
MIMO	Multiple-input and multiple-output
MSE	Mean squared error
NMSE	Normalized mean squared error
OFDM	Orthogonal frequency-division multiplexing
PAPR	Peak-to-average power ratio
PMCW	Phase-modulated continuous wave
PRBS	Pseudorandom binary sequence
PSR	Peak-to-sidelobe ratio
RD	Range–Doppler
RPCA	Robust principal component analysis
SAR	Synthetic aperture radar
SNR	Signal-to-noise ratio
SVD	Singular value decomposition
SVT	Singular value thresholding
